# A nitrite-oxidising bacterium constitutively consumes atmospheric hydrogen

**DOI:** 10.1038/s41396-022-01265-0

**Published:** 2022-06-25

**Authors:** Pok Man Leung, Anne Daebeler, Eleonora Chiri, Iresha Hanchapola, David L. Gillett, Ralf B. Schittenhelm, Holger Daims, Chris Greening

**Affiliations:** 1 Department of Microbiology, Biomedicine Discovery Institute, Monash University, Clayton, VIC 3800 Australia; 2 Division of Microbial Ecology, Centre for Microbiology and Environmental Systems Science, University of Vienna, Vienna, Austria; 3 Soil and Water Research Infrastructure, Biology Centre CAS, Budweis, Czechia; 4 Monash Proteomics and Metabolomics Facility and Department of Biochemistry, Monash Biomedicine Discovery Institute, Monash University, Clayton, VIC 3800 Australia; 5 University of Vienna, The Comammox Research Platform, Vienna, Austria

**Keywords:** Environmental microbiology, Microbial ecology, Metabolism

## Abstract

Chemolithoautotrophic nitrite-oxidising bacteria (NOB) of the genus *Nitrospira* contribute to nitrification in diverse natural environments and engineered systems. *Nitrospira* are thought to be well-adapted to substrate limitation owing to their high affinity for nitrite and capacity to use alternative energy sources. Here, we demonstrate that the canonical nitrite oxidiser *Nitrospira moscoviensis* oxidises hydrogen (H_2_) below atmospheric levels using a high-affinity group 2a nickel-iron hydrogenase [*K*_m(app)_ = 32 nM]. Atmospheric H_2_ oxidation occurred under both nitrite-replete and nitrite-deplete conditions, suggesting low-potential electrons derived from H_2_ oxidation promote nitrite-dependent growth and enable survival during nitrite limitation. Proteomic analyses confirmed the hydrogenase was abundant under both conditions and indicated extensive metabolic changes occur to reduce energy expenditure and growth under nitrite-deplete conditions. Thermodynamic modelling revealed that H_2_ oxidation theoretically generates higher power yield than nitrite oxidation at low substrate concentrations and significantly contributes to growth at elevated nitrite concentrations. Collectively, this study suggests atmospheric H_2_ oxidation enhances the growth and survival of NOB amid variability of nitrite supply, extends the phenomenon of atmospheric H_2_ oxidation to an eighth phylum (Nitrospirota), and reveals unexpected new links between the global hydrogen and nitrogen cycles. Long classified as obligate nitrite oxidisers, our findings suggest H_2_ may primarily support growth and survival of certain NOB in natural environments.

## Introduction

Bacteria of the genus *Nitrospira* are the most widespread group of nitrite-oxidising bacteria. These bacteria are diverse and abundant in many natural environments, as well as wastewater treatment plants and drinking water treatment systems, where they play key roles in nitrification [1]. Reflecting the high standard redox potential of nitrite, chemolithoautotrophic growth on nitrite alone is a challenging lifestyle given little ATP is produced during catabolic processes and reverse electron flow is necessary for anabolic processes. However, it has recently been revealed that nitrite-oxidising *Nitrospira* are more metabolically flexible than previously realised. In addition to mediating aerobic nitrite oxidation via the key enzyme nitrite oxidoreductase, cultured representatives can use ammonia (comammox bacteria), formate, and hydrogen (H_2_) as electron donors for aerobic respiration or (with formate and H_2_) nitrate respiration [2–6] Such flexibility is thought to enhance the growth and survival of *Nitrospira* under different conditions, including amid variability or limitation in nitrite supply.

The model nitrite-oxidising bacterium *Nitrospira moscoviensis* can aerobically grow on H_2_ as its sole energy and electron source in the absence of nitrite, albeit with a lower growth rate than on nitrite [3]. Encoding a group 2a [NiFe]-hydrogenase, this bacterium is predicted to use the high-energy electrons derived from H_2_ oxidation to support both aerobic respiration and carbon fixation through the reverse tricarboxylic acid (rTCA) cycle [3, 7]. Previously, *N*. *moscoviensis* was found to transcribe hydrogenase genes even when grown on nitrite with air in the headspace, but without externally added H_2_ [3, 8]. Based on those results, we speculated that *N. moscoviensis* may be capable of both using H_2_ at elevated concentrations and potentially even scavenging atmospheric H_2_. However, these previous studies did not attempt to measure atmospheric H_2_ uptake or the kinetic parameters of this process.

Consistent with this hypothesis, recent studies have shown atmospheric H_2_ is a desirable energy source for bacteria. While the concentration of atmospheric H_2_ (530 ppbv) is thought to be too low to support growth, this gas nevertheless serves as a dependable lifeline for long-term survival and can be a useful supplement during mixotrophic growth. This reflects its ubiquitous availability, high diffusibility, low activation energy, and high energy yield [9, 10]. Culture-based studies have revealed bacteria from at least seven phyla oxidise atmospheric H_2_, including various organotrophs, lithotrophs, and methanotrophs [11–16]. These bacteria each possess either high- or medium-affinity group 1h, 2a, 1f, or 1l [NiFe]-hydrogenases [16–19] that input electrons into aerobic respiratory chains. Bacterial atmospheric H_2_ oxidation is also biogeochemically significant, contributing to the net loss of three-quarters of the H_2_ removed from the atmosphere each year [10]. In this study, we addressed the hypothesis that nitrite-oxidising bacteria are also capable of oxidising atmospheric H_2_. To do so, we performed kinetic, proteomic, and thermodynamic modelling analyses of aerobic H_2_ oxidation by *N. moscoviensis*, which encodes a group 2a [NiFe]-hydrogenase as its sole H_2_-metabolising enzyme, in the presence and absence of nitrite as the main substrate.

## Methods

### Bacterial strain and growth conditions

*Nitrospira moscoviensis* was maintained in mineral medium at 37 °C with shaking at 100 rpm in the dark, unless otherwise specified. The medium base (per litre water) contained 0.15 g KH_2_PO_4_, 0.05 g MgSO_4_·7H_2_O, 0.5 g NaCl, 0.01 g CaCO_3_, 0.01 g NH_4_Cl, 34.4 μg MnSO_4_∙H_2_O, 50 μg H_3_BO_3_, 70 μg ZnCl_2_, 72.6 μg Na_2_MoO_4_∙2H_2_O, 20 μg CuCl_2_∙2H_2_O, 24 μg NiCl_2_∙6H_2_O, 80 μg CoCl_2_∙6H_2_O, 1 mg FeSO_4_∙7H_2_O, 3 μg Na_2_SeO_3_∙5H_2_O, and 4 μg Na_2_WO_4_∙2H_2_O [3]. The pH of the medium was adjusted to 8.4–8.6 before autoclaving. Filter-sterilised NaNO_2_ was added to the sterile medium at a final concentration of 1 mM. During incubations, nitrite consumption was monitored regularly with Quantofix nitrite indicator strips (Macherey-Nagel). Nitrite was replenished to a concentration of 1 mM when completely consumed until transfer of the culture to fresh medium. Heterotrophic contamination was tested regularly by inoculating 200 μl of the culture onto nutrient agar, 5% R2A agar and nutrient broth at 37 °C, and monitoring for two weeks. Contaminants were not detected in these organic media or through light microscopy of *N. moscoviensis* cultures. Biomass of *N. moscoviensis* was assessed by measuring total cell protein with the enhanced test tube protocol of the BCA protein assay (Sigma-Aldrich).

### Gas chromatography

To determine the ability of *N. moscoviensis* to oxidise H_2_ at sub-atmospheric levels in the presence and absence of nitrite, we prepared nitrite-replete and nitrite-deplete cultures as follows. Cells were harvested by centrifugation (4800 × *g*, 30 min) from an exponentially growing nitrite-oxidising mother culture that was repeatedly fed with 1 mM nitrite. Cell pellets were resuspended in fresh nitrite-free medium and washed twice by centrifuging and resuspending as described above. The absence of nitrite and nitrate in the final washed culture was confirmed by Quantofix nitrite and nitrate indicator strips (Macherey-Nagel). Thirty millilitres of nitrite-free cultures was transferred into sterile 160-ml serum vials sealed with butyl rubber stoppers. One set of triplicate cultures was maintained with 1 mM nitrite as described in the above section (nitrite-replete condition) whereas another set was not supplied with any nitrite (nitrite-deplete condition). Cultures were allowed to adapt to the respective conditions for 48 h. Total cell protein concentrations at the start of assay for nitrite-replete and nitrite-deplete cultures were 1.77 and 1.20 µg ml^−1^, respectively. A medium-only control and a heat-killed control (121 °C, 15 p.s.i. for 20 min) were included in the experiment. For gas chromatography measurements, H_2_ in the air headspace of the vials was amended to give starting mixing ratios of approximately 10 parts per million (ppmv) via 1 % v/v H_2_ (in N_2_; Air Liquide). At each time interval, 2 ml of headspace gas was sampled using a gas-tight syringe and injected into a VICI gas chromatographic machine with a pulsed discharge helium ionisation detector (model TGA-6791-W-4U-2, Valco Instruments Company Inc.) for H_2_ quantification [13]. The machine was calibrated against ultra-pure H_2_ standards down to the limit of quantification (20 ppbv). Calibration mixed gas (10.20 ppmv of H_2_, 10.10 ppmv of CH_4_, 9.95 ppmv of CO in N_2_, Air Liquide) and pressurised air (Air Liquide) with known trace gas concentrations were used as internal reference standards.

### Kinetic analysis

To ensure an accurate determination of H_2_ oxidation kinetics, *N. moscoviensis* kept under nitrite-deplete conditions (as described under the “Gas chromatography” method section) was used to minimise potential biomass deviations caused by nitrite-dependent growth. Triplicate cultures (total cell protein = 30.18 µg each) were incubated independently with approximately 10, 30, 90, 300, and 900 ppmv H_2_ in the vial headspace. Concentrations of H_2_ in the headspace were quantified by gas chromatography at three time points, 0 h, 8 h, and 24 h. Reaction rates of H_2_ consumption were calculated based on the concentration change between the second and third time points to account for any potential lag response of the cultures to the concentrations of H_2_ supplemented and allow sufficient time for equilibration of the headspace H_2_ with the aqueous phase. Michaelis–Menten curves and parameters were estimated using the non-linear fit (Michaelis–Menten, least squares regression) function in GraphPad Prism (version 9.0.0). Dissolved H_2_ was calculated by fitting Henry’s Law as previously described [3] with constants for H_2_ adopted from Sander [20].

### Shotgun proteomics

Triplicate 150 ml nitrite-replete and nitrite-deplete cultures were maintained in Schott bottles with ambient air in the headspace and adapted for 48 h as previously described. Cells were then harvested by centrifugation (4800 × *g*, 30 min, 4 °C), resuspended in phosphate-buffered saline (PBS; 137 mM NaCl, 2.7 mM KCl, 10 mM Na_2_HPO_4_ and 2 mM KH_2_PO_4_, pH 7.4), and centrifuged again. The cell pellets were immediately stored at −20 °C and sent to the Proteomics & Metabolomics Facility in Monash University for analysis. The samples were lysed in SDS lysis buffer (5% w/v sodium dodecyl sulfate, 100 mM HEPES, pH 8.1), heated at 95 °C for 10 min, and then probe-sonicated before measuring the protein concentration using the BCA method. Equivalent amounts of lysed samples were denatured and alkylated by adding TCEP (tris(2-carboxyethyl) phosphine hydrochloride) and CAA (2-chloroacetamide) to a final concentration of 10 mM and 40 mM, respectively, and the mixture was incubated at 55 °C for 15 min. The reduced and alkylated proteins were then immobilised in S-Trap mini columns (Profiti) and sequencing-grade trypsin was added at an enzyme to protein ratio of 1:50 and incubated overnight at 37 °C. Tryptic peptides were sequentially eluted from the columns using (i) 50 mM TEAB, (ii) 0.2% formic acid and (iii) 50% acetonitrile and 0.2% formic acid. The fractions were pooled and concentrated in a vacuum concentrator prior to MS analysis. To ensure an absolute quantification of protein abundance and allow fair comparison across samples, the same amount of peptides was injected into the mass spectrometer for each sample. Using a Dionex UltiMate 3000 RSLCnano system equipped with a Dionex UltiMate 3000 RS autosampler, an Acclaim PepMap RSLC analytical column (75 µm × 50 cm, nanoViper, C18, 2 µm, 100 Å; Thermo Scientific) and an Acclaim PepMap 100 trap column (100 µm × 2 cm, nanoViper, C18, 5 µm, 100 Å; Thermo Scientific), the tryptic peptides were separated by increasing concentrations of 80% acetonitrile (ACN) / 0.1% formic acid at a flow of 250 nl min^−1^ for 158 min and analysed with a QExactive HF mass spectrometer (ThermoFisher Scientific). The instrument was operated in data-dependent acquisition mode to automatically switch between full-scan MS and MS/MS acquisition. Each survey full scan (375–1575 m/z) was acquired with a resolution of 120,000 (at 200 *m*/*z*), an AGC (automatic gain control) target of 3 × 10^6^, and a maximum injection time of 54 ms. Dynamic exclusion was set to 15 s. The 12 most intense multiply charged ions (z ≥ 2) were sequentially isolated and fragmented in the collision cell by higher-energy collisional dissociation (HCD) with a fixed injection time of 54 ms, 30,000 resolution and an AGC target of 2 × 10^5^. The raw data files were analysed with the MaxQuant software suite v1.6.5.0 [21] and its implemented Andromeda search engine [22] to obtain protein identifications and their respective label-free quantification (LFQ) values using standard parameters. Protein abundance across the samples was normalised (median normalisation of protein/peptide intensities). These data were further analysed with LFQ-Analyst [23] as follows. First, contaminant proteins, reverse sequences and proteins identified “only by site” were filtered out. Also removed were proteins only identified by a single peptide and proteins that have not been identified consistently. The LFQ data was converted to log_2_ scale, samples were grouped by conditions and missing values were imputed using the ‘Missing not At Random’ (MNAR) method, which uses random draws from a left-shifted Gaussian distribution of 1.8 StDev (standard deviation) apart with a width of 0.3. Protein-wise linear models combined with empirical Bayes statistics were used for the differential expression analyses. The ‘limma’ package from R Bioconductor was used to generate a list of differentially expressed proteins for each pairwise comparison. A cutoff of the ‘adjusted *p* value’ of 0.05 (Benjamini-Hochberg method) along with a |log_2_ fold change| of 1 was applied to determine significantly differentially abundant proteins in each pairwise comparison.

### Thermodynamic modelling

To estimate to what extent H_2_ and nitrite oxidation contribute to cellular power of *N. moscoviensis* at different substrate levels, we performed thermodynamics modelling to calculate their respective theoretical energy yields according to the consumption kinetics of *N. moscoviensis* (Gibbs free energy per unit time per protein biomass). Power, *P* follows the equation:

1 $$P = v \cdot \Delta G_{{{{{\rm{r}}}}}}$$

where *v* denotes the rate of substrate consumption per protein biomass of *N. moscoviensis* cultures (mol s^−1^ g protein^−1^) and is obtained from the Michaelis–Menten equation of the respective reaction: H_2_ + 0.5 O_2_ → H_2_O (dihydrogen oxidation); NO_2_^−^ + 0.5 O_2_ → NO_3_^−^ (nitrite oxidation). Kinetics parameters for H_2_ oxidation were derived from the current study while those for nitrite oxidation by early stationary phase culture (12–48 h post nitrite depletion) of *N. moscoviensis* grown at 37 °C were obtained from Nowka et al. 2015 [24], where a *V*_max(app)_ of 18,000 µmol h^−1^ g protein^−1^ and *K*_m(app)_ of 9 µM were reported. *ΔG*_*r*_ represents the Gibbs free energy of the reaction at the experimental conditions (J mol^−1^) and follows the equation:

2 $${{\Delta }}G_{{{{{\rm{r}}}}}} = {{\Delta }}G_{{{{{\rm{r}}}}}}^0 + {{{{{\rm{RT}}}}}}\;{{{{{{{\mathrm{ln}}}}}}}}Q_{{{{{\rm{r}}}}}}$$

where *ΔG*_r_^*0*^ denotes the standard Gibbs free energy of the reaction, *Q*_*r*_ denotes the reaction quotient, *R* represents the ideal gas constant, and *T* represents temperature in Kelvin. Values of *ΔG*_*r*_^*0*^ of the H_2_ oxidation and nitrite oxidation were obtained from Thauer et al. (1977) [25]. Values of *Q*_*r*_ for each reaction were calculated using:

3 $$Q_r = {\prod} {a_i^{n_i}}$$

where *a*_*i*_ and *n*_*i*_ denote the concentration of the *i*th species in water and the stoichiometric coefficient of the *i*th species in the reaction of interest, respectively. Gibbs free energy for oxidation of H_2_ and nitrite at atmospheric pressure, an incubation temperature of 37 °C, and various molar ratios of nitrate:nitrite were calculated.

## Results and discussion

### *Nitrospira moscoviensis* oxidises atmospheric H_2_ during growth and persistence using a high-affinity group 2a [NiFe]-hydrogenase

**Fig. 1:**
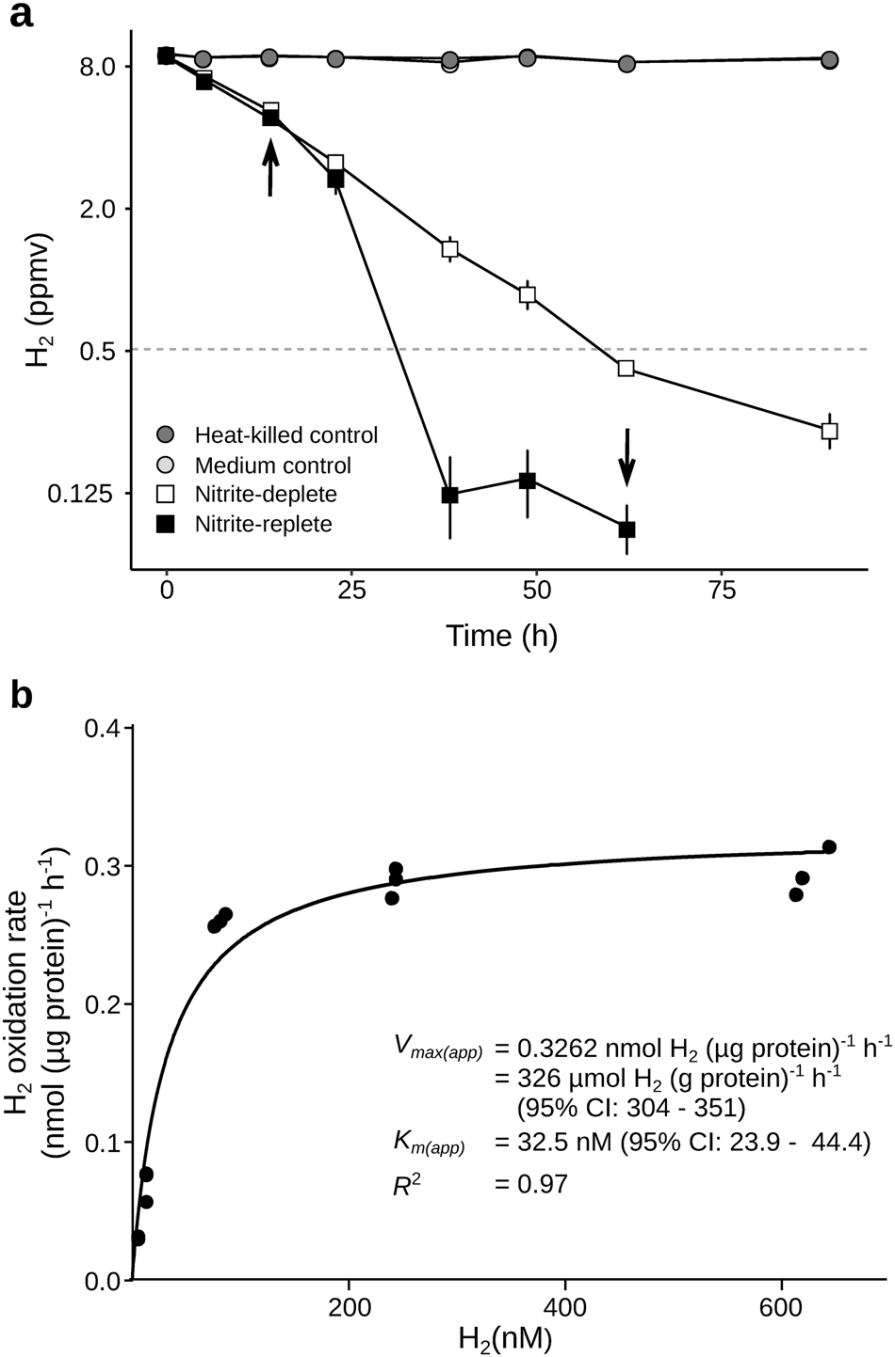
Hydrogen oxidising activities of *Nitrospira moscoviensis* during nitrite-replete and nitrite-deplete conditions. **a** Oxidation of molecular hydrogen (H_2_) to sub-atmospheric levels by *N. moscoviensis* cultures. Error bars show standard errors of three biological replicates, with heat-killed cells and medium-only incubations as negative controls. The mixing ratio of H_2_ is on a logarithmic scale, and the grey dotted line indicates the average atmospheric mixing ratio (0.53 ppmv). Black arrows indicate time points at which nitrite had been completely oxidised and was replenished in the nitrite-replete cultures. **b** Kinetics of H_2_ oxidation by *N. moscoviensis* cells. The curve was fitted and kinetic parameters were calculated based on a Michaelis–Menten non-linear regression model. Note that dissolved H_2_ levels in the culture medium were 0.39 nM H_2_ at atmospheric conditions and at 37 °C.

Hydrogenase activity of *N. moscoviensis* cultures was measured during growth under nitrite-replete and during survival under nitrite-deplete conditions. The bacterium was incubated in closed vials with ambient air headspace supplemented with ~10 ppmv of H_2_, and H_2_ mixing ratios were monitored using gas chromatography. Consistent with our hypothesis, the cultures oxidised H_2_ in a first-order kinetic process to mixing ratios of 98 ppbv (nitrite-replete cultures) and 248 ppbv (nitrite-deplete cultures), which were five and two times below tropospheric H_2_ levels, respectively (Fig. 1a). No decrease of H_2_ was observed in heat-killed and medium-only controls, excluding H_2_ leakage as a source of bias in these experiments. Atmospheric H_2_ oxidation in the phylum Nitrospirota and by nitrifying microorganisms has not been observed earlier. The capacity of *N. moscoviensis* to oxidise atmospheric H_2_ is consistent with the physiology of other bacteria (*Mycobacterium smegmatis*, *Gemmatimonas aurantiaca*, *Acidithiobacillus ferrooxidans* and *Chloroflexus aggregans*) expressing group 2a [NiFe] hydrogenases [14, 26].

Whole-cell kinetic measurements revealed that H_2_ oxidation by *N. moscoviensis* followed Michaelis–Menten-type kinetics (*R*^2^ = 0.97), with a mean apparent half-saturation constant [*K*_m(app)_] of 32.5 nM H_2_ (95% CI: 23.9 to 44.4) (Fig. 1b). The calculated mean maximum oxidation rate [*V*_max(app)_] was 326 µmol H_2_ per g of protein per h (95% CI: 304 to 351) (Fig. 1b). This suggests that *N. moscoviensis* possesses an unusually high-affinity and fast-acting group 2a [NiFe]-hydrogenase adapted for atmospheric H_2_ uptake. Its apparent H_2_ affinity is among the highest of any microorganism reported to date, comparable to the actinobacterium *Streptomyces avermitilis* (*K*_m(app)_ = 39 nM) and the acidobacterium *Pyrinomonas methylaliphatogenes* (*K*_m(app)_ = 35 nM), and in line with the high-affinity activities observed in whole soils (*K*_m(app)_ = 10 to 70 nM) [12, 27, 28].

### Proteome analysis suggests high hydrogenase expression and significant metabolic remodelling following nitrite depletion

**Fig. 2:**
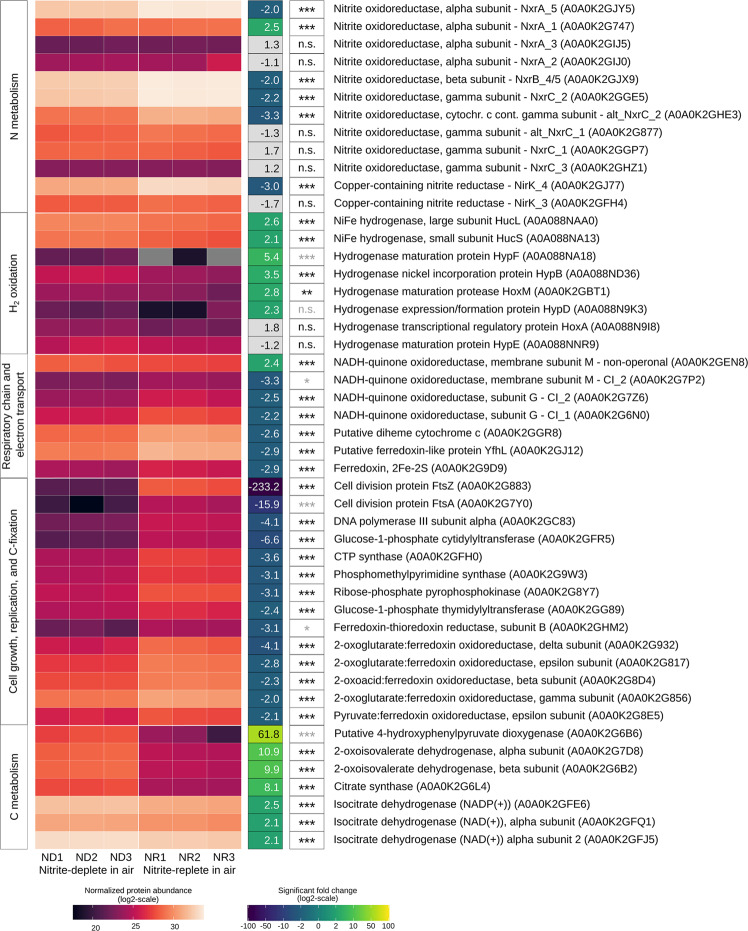
Heatmap of selected *N. moscoviensis* proteins that were differentially abundant under nitrite-deplete versus nitrite-replete conditions. Notably, atmospheric H_2_ was oxidised under both conditions (see Fig. 1a). Normalised protein abundance values were log_2_-transformed, grey colour indicates absence from the proteome. The complete set of untransformed values is listed in Table S1. ND1 to ND3 and NR1 to NR3 are three biological replicates under nitrite-deplete and nitrite-replete conditions, respectively. Fold changes under nitrite-deplete conditions and the corresponding significance values (adj. *p* value ≤ 0.001, ***; ≤0.01, **; ≤0.05, *) are shown in the two boxes next to each protein. Yellow-green and blue colour indicate significant positive and negative fold changes, respectively, and grey asterisks indicate that abundance values were imputed. Select low-abundance proteins of interest were included despite being not statistically significant. Depicted proteins are sorted based on their functional category as displayed on the left side. UniProtKB accession numbers are indicated in parentheses. CI_1, 2M-type NADH-quinone oxidoreductase 1; CI_2, NADH-quinone oxidoreductase 2; nomenclature of nitrite oxidoreductase subunits according to Mundinger et al. [8].

**Fig. 3:**
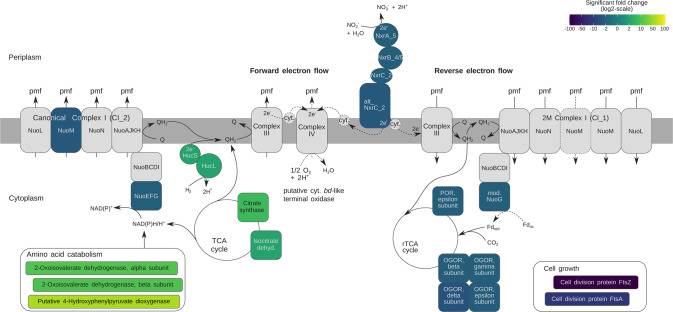
Genome-based model of energy and CO_2_ fixation metabolism in *N. moscoviensis*, showing significant fold changes (min. twofold) in the relative abundance levels of selected proteins under nitrite-deplete conditions compared to nitrite-replete conditions. The significant fold change level is indicated for each protein by colour, with grey indicating no significant fold change of at least 2-fold. Quinone reduction by the hydrogenase (Huc) is shown in the context of respiration, but the resulting quinol may also fuel reverse electron flow as outlined in the main text. Dashed lines indicate features and electron flow pathways awaiting experimental confirmation in *Nitrospira* or other organisms. Respiratory complexes are labelled with roman numerals. Cyt. cytochrome; Fd ferredoxin; OGOR 2-Oxoglutarate-ferredoxin oxidoreductase; POR pyruvate-ferredoxin oxidoreductase; Q quinone. For further details, refer to the main text, Fig. 2, and Supplementary Table S1.

To gain a holistic perspective of the *N. moscoviensis* metabolism, we compared shotgun proteomes from triplicate cultures grown under nitrite-deplete versus nitrite-replete conditions. Whole-cell proteome analysis resulted in the detection of 2244 of the 4733 non-identical proteins (57.8%) encoded in the *N. moscoviensis* genome (Table S1). The proteome significantly changed under the nitrite-deplete condition, with 151 proteins more abundant and 293 proteins less abundant by at least twofold (*p* < 0.05; Table S1). Nitrite oxidoreductase and group 2a [NiFe] hydrogenase subunits were highly abundant in both conditions, though appear to be differentially regulated (Figs. 2, 3 and Table S1). The *N. moscoviensis* genome encodes multiple copies of the nitrite oxidoreductase (Nxr) catalytic subunit NxrA, and beta and gamma subunits NxrB and NxrC, respectively (see Supplementary Note for a discussion of the Nxr isoforms). One paralog each of NxrA, NxrB, and NxrC were among the ten most abundant proteins detected under nitrite-replete conditions, but their expression decreased by twofold (*p* < 0.001) following nitrite starvation (Fig. 2, Fig. 3, Table S1, Supplementary Note). Conversely, the group 2a [NiFe]-hydrogenase catalytic subunit (HucL) increased 2.6-fold during starvation (*p* < 0.001) (Figs. 2 and 3). We also detected most other hydrogenase-related structural, maturation, and accessory proteins whose gene transcription had earlier been observed during growth on H_2_, with the exceptions of HypC, HypA, and UreH [3]. Altogether, this suggests that the hydrogenase appears to be constitutively expressed and slightly upregulated by *N. moscoviensis* under nitrite-deplete starvation conditions. These observations parallel those made regarding the group 2a [NiFe]-hydrogenase of *M. smegmatis*, but contrast with *G. aurantiaca*, *A. ferrooxidans* and *C. aggregans* for which hydrogenase expression and activity peaked during growth [14, 19].

Extensive other changes in the *N. moscoviensis* proteome were evident under nitrite-deplete conditions. Production of various proteins associated with biomass formation and growth decreased, including proteins responsible for cell division, DNA synthesis and replication, carbon fixation via the rTCA cycle, and amino acid synthesis (Figs. 2, 3 and Table S1). *N. moscoviensis* also potentially generates additional reductant in nitrite-deplete conditions by catabolizing amino acid reserves (see Supplementary Note). Moreover, enzymes of the TCA cycle (citrate synthase and two forms of isocitrate dehydrogenase) were 8.1-, 2.5-, and 2.1-fold (*p* < 0.001) more abundant under nitrite-deplete conditions (Figs. 2, 3 and Table S1). The increased production of citrate synthase, a key enzyme of the oxidative TCA cycle, further indicates the increased energy harvesting from storage compounds. Rather unexpectedly, the relative abundance of proteins related to glycogen mobilisation did not significantly vary (e.g., glycogen synthase GlgA, 1,4-alpha-glucan branching enzyme GlgB, glycogen debranching enzyme GlgX; Table S1). This observation potentially suggests that *N. moscoviensis* uses other compounds (e.g., amino acids) prior to glycogen under starvation, or that glycogen reserves play a role other than for persistence in this organism. It should be noted that, while glycogen metabolism genes are found in most *Nitrospira* genomes [3, 29], biochemical approaches are required to validate that glycogen is synthesised as a storage compound and resolve the factors controlling its synthesis and degradation.

Finally, the proteome profiles indicate a broader remodelling of the respiratory chain and differential use of electron transport proteins. *N. moscoviensis* encodes three forms of complex I (NADH-quinone oxidoreductase) [3], designated as Cl_1 to Cl_3 [8]. Subunits M and G of CI_2 and the G subunit of CI_1 were less abundant (*p* < 0.05, 0.001 and 0.001, respectively) in the nitrite-deplete condition (Figs. 2 and 3 and Table S1). Both forms of complex I are likely important for proton motive force-driven reverse electron transport under nitrite-oxidising conditions. CI_2 may catalyse NADH production [8], whereas the 2M-type CI_1 might reduce ferredoxins for the rTCA cycle, interacting with ferredoxin *via* its G subunit [7, 30]. Thus, we conclude that in the nitrite-deplete condition, proteins connected to reverse electron transport and ferredoxin reduction were less abundant, which is consistent with the same findings regarding rTCA cycle enzymes (see above). Further analysis of the proteomic data is provided in the Supplementary Note.

### Energetic contribution of hydrogen and nitrite oxidation is differentiated by consumption kinetics and substrate availability in the environment

Altogether, H_2_ oxidation is a highly advantageous metabolism to the growth and persistence of *N. moscoviensis*, as reflected by (i) the observed high-affinity and fast-acting kinetics, (ii) the constitutive expression of hydrogenases suggested by proteomics, and (iii) the relatively low-potential electrons derived from this process (−420 mV) compared to nitrite oxidation (+420 mV) [31]. To understand the relative contribution of H_2_ and nitrite oxidation to *N. moscoviensis* energetics, we performed thermodynamic modelling to calculate the theoretical power yield of both reactions at a wide range of environmentally relevant substrate concentrations based on their respective consumption kinetics (Materials and Methods). A *V*_max(app)_ of 18,000 µmol per g of protein per h and *K*_m(app)_ of 9 µM were previously reported for nitrite oxidation by nitrite-deplete early stationary phase cultures of *N. moscoviensis* grown at 37 °C [24], which is compatible with the culture conditions used in the current study.

**Fig. 4:**
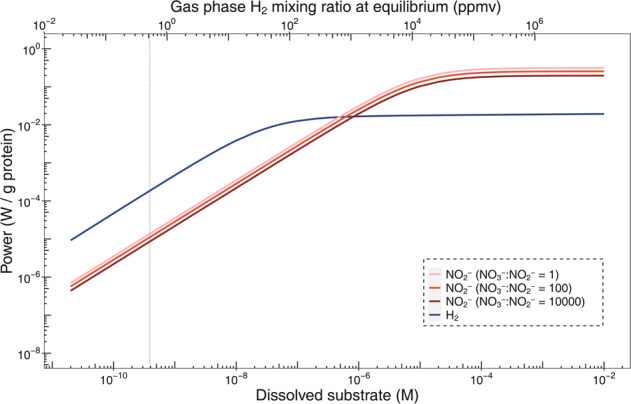
Thermodynamic modelling of the power yield from H_2_ and nitrite oxidation at various substrate concentrations based on the Michaelis–Menten kinetics of the reactions by *N. moscoviensis* cultures. The power yields for nitrite oxidation at nitrate:nitrite molar ratios 1, 100, and 10,000 are shown. The dotted vertical line indicates the atmospheric H_2_ concentration.

Based on the thermodynamics modelling, at sub-micromolar substrate concentrations typical of natural soil and aquatic ecosystems [32, 33], the rate of H_2_ oxidation consistently yields more power for *N. moscoviensis* than nitrite oxidation (Fig. 4). At atmospheric levels of H_2_, the power yield of H_2_ oxidation is 1.9 × 10^−4 ^W per g protein, which is an order of magnitude higher than derived from oxidation of equivalent concentrations of nitrite. These observations are consistent with the hypothesis that atmospheric H_2_ oxidation can support persistence of *Nitrospira* amid nitrite starvation. Above micromolar substrate concentrations, for example as will likely occur in engineered systems such as wastewater treatment plants [34], the hydrogenase reaches saturation while the rate and power of nitrite oxidation increases with nitrite availability up to micromolar ranges. These observations are concordant with our previous inferences that the logarithmic protein increase of *N. moscoviensis* grown on nitrite (~1 mM) is due to higher maximum reaction rates, while the linear protein increase when grown on elevated H_2_ alone (70% headspace H_2_) is due to kinetic constraints [3]. These observations also suggest that the high maximal nitrite consumption rate is one of the defining features of nitrite-oxidising *Nitrospira*. Nevertheless, H_2_ oxidation appears to be beneficial amid elevated nitrite supply given both reactions co-occur during growth of *N. moscoviensis*; in addition to generating additional power from catabolic processes from H_2_ (Fig. 4), it is probable that the bacterium preferentially uses the relatively low-potential electrons derived from H_2_ compared to nitrite for CO_2_ fixation through the rTCA cycle (Fig. 3).

## Conclusions

In conclusion, this study shows that *N. moscoviensis* can efficiently oxidise atmospheric H_2_ with a high-affinity hydrogenase, indicating that the environmentally widespread *Nitrospira* bacteria likely contribute to atmospheric H_2_ uptake in nature. Together with the previously demonstrated capabilities of *N. moscoviensis* to grow chemolithoautotrophically on elevated H_2_ concentrations and chemoorganoautotrophically on formate without nitrite [3, 4], the use of atmospheric H_2_ adds to the impressive metabolic versatility of this organism. Far from the classical view that *Nitrospira* are obligate nitrite oxidisers [35–37], our results suggest that this bacterium may predominantly use H_2_ to meet cellular power needs in naturally substrate-limited soils and waters.

Additionally, H_2_ oxidation likely enables *N. moscoviensis* to adapt to a wide range of nitrite concentrations. Our results suggest that atmospheric H_2_ serves as an alternative source of energy and electrons during nitrite starvation, likely improving the capacity of *N. moscoviensis* to persist during periods of low substrate availability. Under these conditions, *N. moscoviensis* remodels its metabolism extensively to reduce energy expenditure and growth, in addition to scavenging H_2_ from air. The oxidation of H_2_ is likely to yield more power than nitrite oxidation at low (sub-micromolar) substrate concentrations typical of natural environments, whereas nitrite oxidation has a dominant role at elevated concentrations typical of engineered systems or in the direct vicinity of ammonia-oxidising microorganisms [38]. Under nitrite-replete conditions, the use of atmospheric H_2_ likely still serves as a convenient source of electrons for CO_2_ fixation, reducing the need for energetically expensive reverse electron transport from nitrite to the low-potential ferredoxins of the rTCA cycle. Through this combination of strategies, *N. moscoviensis* is likely to be able to adapt to a wide range of environments and spatio-temporal variations in substrate availability.

Further work is needed to determine if these findings extend to other NOB. The genomes of many other NOB (with the exception of *Nitrolancea* in the phylum Chloroflexi) [1, 39] lack known high-affinity hydrogenases, suggesting they are more metabolically constrained than *N. moscoviensis* [1, 40]. However, other *Nitrospira* genomes harbouring Huc-type hydrogenases have been reported from a range of environments, including alkaline lakes [41] and activated sludge [42]. Hence, atmospheric H_2_ utilisation may confer a selective advantage over other nitrite oxidisers and could contribute to the frequently observed predominance of *Nitrospira* in natural and engineered nitrifying microbial communities.

## Supplementary information

Supplementary Note

Supplementary Table S1

## Data Availability

The raw datasets for kinetic and proteome analyses generated during the current study are publicly available on figshare (10.26180/19930124.v2 and 10.26180/19930127.v1).
