# Supplementary material for: A nitrite-oxidising bacterium constitutively consumes atmospheric hydrogen
Source: ISME J. 2022 Jun 25;16(9):2213–9. doi: 10.1038/s41396-022-01265-0 (PMC9381531; doi:10.1038/s41396-022-01265-0)
Supplement: Supplementary file 1 — Supplementary Note [file 41396_2022_1265_MOESM1_ESM.pdf]

Supplementary Material: A nitrite-oxidising bacterium constitutively consumes atmospheric hydrogen

Supplementary Tables

Table S1. Differential protein expression analysis of *N. moscoviensis* grown under nitrite-deplete and nitrite-replete conditions in air.

Supplementary Note

Nitrite oxidoreductase (Nxr), the key enzyme for nitrite oxidation in *Nitrospira*, consists of at least three subunits: alpha (NxrA), beta (NxrB), and gamma (NxrC) [1]. The *N. moscoviensis* genome encodes four paralogous alpha, four beta, and five gamma NXR subunits. A fifth set alpha and beta subunits were present in the originally sequenced genome [2] but have been lost from the laboratory strain after deletion of a genomic region [3]. In the proteomes, we detected four alpha, one beta, and five gamma subunits of NXR. Their protein expression was consistent with transcriptomic data from *N. moscoviensis* reported by Mundiger *et al.* [3]. Among these, the three subunits NxrA_5, NxrB_4/5, and NxrC_2 were among the 10 most abundant detected proteins in nitrite-replete conditions (nomenclature as in [3]). They were significantly downregulated (~2×) in the nitrite-deplete cultures ($p < 0.001$; Fig. 2, Table S1). Two of the gamma subunits (alt_NxrC_1 and alt_NxrC_2) contain a predicted C-terminal transmembrane helix next to signal peptides for translocation into the periplasm *via* the Sec pathway, suggesting a membrane-anchored localization. The other three gamma subunits only contain the signal peptides and are likely soluble in the periplasm [3]. In line with previously reported transcriptomic and genomic analyses of *N. moscoviensis*, the NxrC_2 gamma subunit (lacking a transmembrane helix) showed the highest abundance in the proteomes, which was comparable to the most abundant alpha and beta subunits. Thus, the largest fraction of Nxr enzyme in *N. moscoviensis* is likely soluble in the periplasm. It may interact there with the membrane-anchored, also highly expressed (Table S1) alt_NxrC_1 and

alt_NxrC_2 subunits, for example to transfer electrons from nitrite into the membrane-bound respiratory chain [1, 3].

Methylisocitrate lyase, which functions in the methylcitrate cycle (a modified version of the TCA cycle) allowing for the catabolism of propionic acid without a requirement for cobalamin (Vitamin B12), was upregulated ~3× ($p < 0.001$, Table S1) in the nitrite-deplete condition. Since many proteins involved in the energy-expensive biosynthesis of cobalamin were strongly downregulated under nitrite depletion (Table S1), we assume that *N. moscoviensis* uses the methylcitrate cycle for the catabolism of propionate, or of other fatty-acids with an odd number of carbon atoms via beta-oxidation. Since we did not detect any NADH-quinone oxidoreductase CI_3 subunits in the proteomes, we assume that this form of respiratory complex I in *N. moscoviensis* may not have a function under the tested conditions.

N. moscoviensis potentially generates additional reductant in nitrite-deplete conditions by catabolising amino acid reserves. A putative 4-hydroxyphenylpyruvate dioxygenase, which catalyses the second step in the catabolism of tyrosine, and a putative branched-chain amino-acid dehydrogenase (2-oxoisovalerate dehydrogenase) were among the ten most differentially abundant proteins, upregulated by 62- and 10-fold respectively ($p < 0.001$) (Fig. 2, Fig. 3, Table S1). No specific amino acid storage is known in *Nitrospira*, but we cannot exclude this possibility given the large number of hypothetical proteins in *Nitrospira* genomes. Moreover, it is possible that *N. moscoviensis* is capable of recycling necromass produced under starvation, including branched-chain amino acids.

Supplementary References

1. H. Daims, S. Lücker, M. Wagner, A new perspective on microbes formerly known as nitrite-oxidizing bacteria. *Trends Microbiol.* **24**, 699–712 (2016).

2. H. Koch, *et al.*, Growth of nitrite-oxidizing bacteria by aerobic hydrogen oxidation. *Science* **345**, 1052–1054 (2014).
3. A. B. Mundinger, C. E. Lawson, M. S. M. Jetten, H. Koch, S. Lücker, Cultivation and transcriptional analysis of a canonical *Nitrospira* under stable growth conditions. *Front. Microbiol.* **10** (2019).
